# What do we know about perioperative hypersensitivity reactions and what can we do to improve perioperative safety?

**DOI:** 10.1080/07853890.2021.1976818

**Published:** 2021-10-11

**Authors:** Urszula Kosciuczuk, Pawel Knapp

**Affiliations:** a Department of Anaesthesiology and Intensive Therapy, Medical University of Bialystok, Białystok, Poland; b Department of Gynecology and Gynecological Oncology, Medical University of Bialystok, Białystok, Poland

**Keywords:** Antibiotics, chlorsuccinylocholine, general anaesthesia, rocuronium

## Abstract

Hypersensitivity reactions are an important aspect of perioperative care and are a crucial interdisciplinary issue in anaesthesiological practice, as well as allergological and laboratory diagnostics. This phenomenon was observed as early as the 1980s and 1990s in Western European countries, and knowledge on this subject has grown significantly over time. Although hypersensitivity reactions are not frequent events (the incidence of perioperative hypersensitivity reactions ranges from 1:386 to 1:13 000 procedures, with higher frequency − 1 per 6500 general anaesthesias with neuromuscular blocking agents administrations), their courses are unfortunately serious and life-threatening. It should also be noted that there is no information regarding the occurrence of perioperative hypersensitivity reactions in many countries. Hence, global assessment of the problem is underestimated. The primary source of actual knowledge comes from epidemiological studies, which indicate an increasing frequency of hypersensitivity reaction occurrence and changes in aetiological factors. The first report from France (1984 to 1989) described two main causes – neuromuscular blocking agents and hypnotic agents. The following years confirmed an increase in perioperative hypersensitivity reactions associated with latex and antibiotics. The most recent data from the National Audit Project 6 indicated increased participation of antibiotics, chlorhexidine, and contrast agents. The results of epidemiological analyses are the basis of medical management guidelines and practice modification. Thanks to the activity of many organisations monitoring the intensity and nature of perioperative hypersensitivity reactions, guidelines for diagnostics and management have been developed. This article presents the results of numerous studies, including the first and the most recent, from various geographical regions. The clinical significance, pathogenesis mechanisms are also discussed. This publication also presents important directions for further scientific and epidemiological research on perioperative hypersensitivity reactions.

**Key messages**
- The incidence of perioperative hypersensitivity reactions ranges from 1:386 to 1:13 000 procedures, with higher frequency – 1 per 6500 general anaesthesias with neuromuscular blocking agents administrations.
- Reactions may occur during the first episode of anaesthesia, most frequently in the induction of general anaesthesia, and much less frequently during postoperative follow-up.
- The first reports of perioperative hypersensitivity reaction come from the 1990s, and knowledge on this subject has grown significantly over time.
- In many countries, multidisciplinary teams and organisations have been established to identify, monitor the occurrence of this phenomenon, and have set the directions of medical activities and have changed the rules and recommendations.
- There is no information about the occurrence of perioperative hypersensitivity reactions in many countries, and global assessment of the problem is underestimated. Additionally, there is a great need to develop a system to monitor their occurrence in other countries.
- The long-term epidemiologic studies have demonstrated variability in pharmacologic triggers. However, the main pharmacological substances (antibiotics, muscle relaxants, disinfectans, contrast agents) are related to aspects of patient safety during anaesthesia.

## Introduction

The perioperative (periprocedural) hypersensitivity reactions are an important and multidisciplinary (epidemiological, surgical, anaesthesiological, allergological) topic [1–3]. It is a crucial aspect of perioperative safety, and knowledge on this subject has grown in recent decades.

In many countries, multidisciplinary teams and organisations have been established to identify, monitor the occurrence of this phenomenon, and have set the directions of medical activities and have changed the rules and recommendations. The Perioperative Anaphylactoid Reaction Study Group (GERAP) in France, the Drug Allergy Committee of Spanish Society of Allergy and Clinical Immunology and the Spanish Anaesthesia Society, the Australian and New Zealand Anaesthetic Allergy Group, the European Academy of Allergy and Clinical Immunology (EAACI), and the European Network for Drug Allergy (ENDA) have published epidemiological reports. Moreover, these organisations have formed guidelines to improve diagnostic methods and medical treatment [4–6]. In 2018, the NAP 6 report was published, containing valuable epidemiological and clinical information. In 2019, an initiative of the British Journal of Anaesthesia established the International Suspected Perioperative Allergic Reaction working group (ISPAR) to assess the actual incidence of perioperative hypersensitive reactions and to develop international guidelines [7,8].

Pubmed and Ovid databases were used to present the topic, excluding abstracts and case reports. Publications from the period 1990 to 2020 were evaluated.

## Epidemiology

The first articles indicating the significance of this problem concern observations in the 1980s and 1990s and in the beginning of the twenty-first century in France and Scandinavia [9–18]. The significance of the problem has been especially noted in Scandinavian countries, Western Europe, Australia, and New Zealand [19–21].

The incidence of hypersensitivity adverse reactions has been determined to range from 1:386 to 1:13 000 procedures, with higher frequency − 1 per 6500 general anaesthesias with neuromuscular blocking agents administrations. It was translated into 1 case per 7 years of professional practice [7,8,20].

According to nomenclature guidelines, it is reasonable to use the following terms to describe the severity. Hypersensitivity event refers to an unexpected, abnormal, moderate, or mild reaction in response to exposure to a factor tolerated by healthy individuals. The term anaphylaxis indicates a severe, sudden, progressive, general health- and life-threatening situation caused by exposure to external factors. The Ring-Messner scale (1977) is the most commonly used to describe the clinical course of hypersensitivity reactions. Grade 1 means general skin symptoms (itching, urticaria, flushing, angioedema), Grade 2- with additional respiratory and cardiovascular symptoms (dyspnoea, rhinorrhea, tachycardia, hypotension, laryngeal oedema, bronchospasm, cyanosis, cardiac shock), followed by Grade 4 with respiratory and cardiac arrest.

The most recent observational-epidemiologic study (NAP 6) describes that hypersensitivity reactions have a clinical course corresponding to Grade 3 in 51% of cases with hypotension or bronchospasm and Grade 4 requiring cardiopulmonary resuscitation in 45% of cases. Additionally, tachycardia, flushing or non-urticaria rash, cyanosis or oxygen desaturation, decrease or loss of end-tidal carbon dioxide trace are common symptoms that occurred in 46%, 56%, 41%, 30% of cases, respectively.

The incidence of perioperative or periprocedural anaphylaxis in the United States is 1: 6 531 procedures (15 cases per 100 000 procedures), with a mortality of 1 in 191 652 procedures. The authors presented that periprocedural anaphylaxis had increased mortality compared with non-anaphylaxis complicated procedures − 3.4% vs. 1.4%, (*p* < 0.001), respectively [22]. The most actual epidemiological data from Europe presents the incidence of perioperative death from anaphylaxis of 1 in 313 000 and a per–case mortality rate of 1 in 26.6 cases [7,8,23].

## Causative agents

The first epidemiological report from France in the period 1984 to 1989 described 821 perioperative hypersensitivity reactions during anaesthesia, and the two main causes were neuromuscular blocking agents (81%) and hypnotic agents (11%). Other factors included opioids (3%), antibiotics (2%), latex (0.5%), and colloids (0.5%) [9]. The next report indicated similar causes, while 54% of hypersensitivity events were connected to neuromuscular blocking agent administration resulting from the use of succinylcholine, followed by vecuronium (15%) and benzodiazepines (9%) [10]. An epidemiological report from 1990 to 1991 indicated increase in reactions related to the use of latex increased to 12%, and hypnotics to 3%. Among muscle relaxants, hypersensitivity reactions were still most often associated with the use of succinylcholine (43%), vecuronium (37%), pancuronium (13%), and atracurium (6%) [11]. The first changes in epidemiological factors of perioperative hypersensitivity reactions were noted in a report from France in 1996. Reduced incidence of hypersensitivity reactions to muscle relaxants (59%) was presented, with a constant value for the use of benzodiazepines, an increased incidence of these reactions for latex (19%), hypnotic agents (5%), opioids (up to 3%), plasma substitutes (up to 3%), and antibiotics (up to 3%) [12]. Subsequent epidemiological reports indicated a significantly increasing participation of antibiotics [13]. The following years of epidemiological observations confirmed a further increase in the frequency of perioperative hypersensitivity reactions associated with the use of latex-containing products (22% of cases) and antibiotics (14%) [14].

Ninth consecutive national surveys from France (1984 to 2007) reported decreased frequencies of hypersensitivity reactions connected to neuromuscular blocking agents (from 81 to 47%) and hypnotic agents (from 11% to 1.1) with increased frequencies in latex (from 0.5 to 20%), antibiotics (from 2% to 18%), and colloids (from 0.5% to 2.3%), while opioid causes remained stable [9,15–17].

Comparable epidemiological data were presented from observations in Norway − 66% of cases were connected to the use of neuromuscular blocking agents – succinylcholine (36%), rocuronium (20%), vecuronium (7%), and latex (3%) [18,24]. In contrast, Danish researchers reported that allergologic diagnostics had positive skin tests for various substances in almost 58% of cases of perioperative hypersensitivity reactions, with very few positive diagnostics for muscle relaxants. Moreover, antibiotics, especially penicillins, latex, and hypnotics were the main etiologic agents [25].

Spanish researchers indicated that muscle relaxants are the dominant group of triggers, accounting for approximately 46%, while the role of latex and intravenous anaesthetics (propofol) was also significant (28% and 14%, respectively). Moreover, the period during anaesthesia induction showed the highest risk of their occurrence (50% of cases) [26,27]. In another study in a Spanish population, an increased incidence of hypersensitivity reactions connected to antibiotics (beta-lactam, vancomycin, and ciprofloxacin) was observed [28].

Epidemiological studies from Australia and New Zealand reported a similar situation with the common causes of perioperative hypersensitivity reactions resulting from the use of muscle relaxants, with the dominant role for rocuronium and succinylcholine (56%, and 21%, respectively), while a less prominent role was observed for vecuronium (11%), atracurium (9%), and mivacurium (3%) [29–32].

The authors noticed different epidemiological data in an American population. Antibiotics, especially penicillins and cephalosporins, were of the greatest importance in the development of hypersensitivity reactions during the perioperative period, with up to 50% of cases, while neuromuscular blocking agents accounted for only approximately 10% of aetiological factors [33]. The incidence of latex allergic reactions was 11%. Among antibiotics, the most common hypersensitivity reactions were associated with the use of cefazolin in 46% of cases [33–38].

In the first study from Southeast Asia, it was presented that the aetiological factors underlying postoperative hypersensitivity reactions were identified in 57% of cases, among which antibiotics – cephalosporins (18%) and penicillins (6%), muscle relaxants – most often atracurium (12%) and succinylcholine (6%), opioid analgesics – morphine (12%), and disinfectants – chlorhexidine (6%) were common [39].

The most recent data were published from the United Kingdom. The NAP 6 project indicated an incidence of perioperative hypersensitivity reaction of 1:11 752 procedures. Antibiotics were the dominant factor, followed by neuromuscular blocking agents, disinfectants (chlorhexidine) and intraoperative contrast agents (Patent Blue dye). There were no reactions associated with the use of latex-containing products [7,8,23].

Antibiotics, drugs, general anaesthetics, contrast agents, and disinfectants are the main etiologic agents of perioperative hypersensitivity reactions. However, in nearly 50% of cases, it is not possible to identify the etiologic factors, which has great implications for secondary prevention. The epidemiological data of the main causative agents are presented in Table 1. The incidence of hypersensitivity reactions connected with neuromuscular blocking agents is presented in Table 2.

**Table 1.** Main causative agents of perioperative hypersensitivity events in several studies.

Laxenaire et al.	1997–1998	France	*n = 467*	Neuromuscular blocking agents-69.2%, Latex-12.1%, Antibiotics-8.0%, Hypnotics-3.7%, Opioids-1.4%, Colloids-2.7%
Mertes et al.	1999–2000	France	*n = 789*	Neuromuscular blocking agents-58.2%, Latex-16.7%, Antibiotics-15.1%, Hypnotics-3.4%, Opioids-1.3%, Colloids-4.0%
Dong et al.	2005–2007	France	*n = 1253*	Neuromuscular blocking agents-47.4%, Latex-20%, Antibiotics-18.1%, Hypnotics-1.1%, Opioids-2.2%, Colloids-2.3%
Ebo et al.	2001–2018	Belgium	*n = 568*	Neuromuscular blocking agents-30.9%, Latex-13.5%, Antibiotics-10.5%, Chlorhexidine −7.7%
Iammatteo et al.	2009–2017	USA	*n = 34*	Hypnotics-38%, Neuromuscular blocking agents-26%, Beta lactams-14%, Opioids-8%, Local anaesthetics-6%, Latex-5%, Ondansetron-3%
Meng et al.	2013–2016	United Kingdom	*n = 31*	Antibiotics-52.3%, Neuromuscular blocking agents-38.1%, Morphine-4.8%, Colloids-4.8%
Harper et al.	2018	United Kingdom	*n = 266*	Antibiotics-47.2%, Neuromuscular blocking agents-32.6%, Chlorhexidine-9%, Patent blue dye-4.5%

Authors, period, country, and the number of cases are presented.

**Table 2.** The incidence of neuromuscular blocking agents perioperative hypersensitivity events in several studies.

Sadleir et al.	2002–2011	Australia	*n = 80*	8.0:100 000 administrations over the 10 yr period for rocuronium 2.8:100 000 administrations over the 10 yr period for vecuronium 4.01:100 000 administrations over the 10 yr period for atracurium
Reedy et al^]^	2006–2012	New Zealand	*n = 21*	1:2 079 administrations for succinylocholine 1: 2 498 administrations for rocuronium 1: 22 450 administrations for atracurium
Harper et al.	2018	United Kingdom	*n = 266*	1: 19 070 administrations for neuromuscular blocking agents 1: 9 006 administrations for succinylcholine 1: 17 002 administrations for rocuronium 1: 24 111 administrations for atracurium

Authors, period, country, and the number of cases are presented.

The results of epidemiological analyses have set the directions of medical activities and have changed the rules and recommendations [40–43]. The NAP 6 project reported that 38% of anaesthesiologists believed neuromuscular blocking agents to be primary causative agents, and 30% of them avoid succinylcholine and rocuronium for this reason [7,8]. The Australian and European Urological Society suggested avoiding chlorhexidine use in urethral gels. Due to the increasing share of disinfectants and sensitisation with the use of pholcodine, the authors indicate the need to increase awareness of this phenomenon in medical activity, as well as to monitor pholcodine availability. The Australian and New Zealand Anaesthetic Allergy Group and Norwegian Network for Anaphylaxis during Anaesthesia have restricted pholcodine prescriptions and general availability. Epidemiological data obtained after 3 years of limitations on the pholcodine availability in Norway presented a significant reduction in sensitisation state. The concentration of serum antibodies was reduced from 11% to 2.7% for pholcodine, from 3.7% to 0.3% for succinylcholine, and from 10% to 1.3% for morphine, respectively. Moreover, it was associated with a reduced number of reported hypersensitivity reactions to muscle relaxants from 56 cases in 2007 to 34 cases in 2009, also. Although latex hypersensitivity reactions have not been reported in epidemiological studies in recent years, there is still a need to monitor [44].

## Pathomechanism

Perioperative hypersensitivity reactions are mediated by cellular and humoral immune responses or by a nonimmune process triggered by the direct activation of mast cells [45–47].

The chemical structure of allergens and the induction of sensitisation play an important role in the immunological mechanism. The sensitisation phenomenon is associated with the formation of a cellular and humoral response after the first contact with an allergen, which leads to the synthesis of specific immunoglobulin E (IgE) complexed with high-affinity FcɛRI receptors located on the mast cell membrane. Encountering the allergen a second time causes the formation of a permanent antigen, IgE–FcɛRI junction, and then, by changing the conformation of the membrane receptor, activation of mast cells occurs. The formation of newly synthesised mediators, as well as the degranulation and release of stored mediators, is the final effect [48,49]. In animal models, immune-triggered hypersensitivity and anaphylactic reactions have also been reported with the use of IgG when using monoclonal antibodies of human origin [45].

Knowledge in recent decades has highlighted additional information related to the non-immune mechanisms of hypersensitivity reactions. The mechanisms of non-immune activation of mast cells result from the interaction of anaphylatoxins C3a and C5a with specific receptors. Recent publications have explained that neuromuscular blocking agents (atracurium, rocuronium) and antibiotics (ciprofloxacin, levofloxacin) activate mast cells through an Ig E-independent mechanism *via* the MRGPRX 2 receptor (MAS-related G protein-coupled receptor member X2). Another non-immunological hypersensitivity reaction mechanism is associated with the use of lysosomal chemicals called CARPA (C-activation-related pseudoallergy). This mechanism has been described using the lysosomal form of doxorubicin and paclitaxel [45,50–54].

Mast cells are the major effector cells for both immune and non-immune hypersensitivity reactions. In epidemiological observations, hypersensitivity reactions occur more frequently in the immune mechanism and account for approximately 60% of cases [10,11,28,33,37].

## Triggering factors

The greatest risk of perioperative hypersensitivity reactions is associated with the induction phase of general anaesthesia and the use of muscle relaxants, concerning surgical procedures in general surgery, gynaecology, and obstetrics, maxillary surgery, and laryngology [22,55]. Reactions occur most frequently intraoperatively, during the induction and maintenance phase of general anaesthesia (80%), and much less frequently occurring during postoperative follow-up (Post-Anaesthesia Care Unit or Surgery Department) (20%) [7,8,27,32]. The most common hypersensitivity reactions occurred within 5 min of induction of general anaesthesia (86% of cases), while those between 5–10 min and 10–20 min, accounted for only 4% of cases [18,24,28].

Many authors have indicated that female sex is the main predisposing factor for these complications, and the ratio of the incidence of perioperative hypersensitivity reactions is 3:1 female:male. Patient age has also been found to be an important risk factor. Hypersensitivity reactions to latex exposure have been reported to occur more frequently in childhood. In the adult population, hypersensitivity reactions were more common in women approximately 40 years of age and in men over 50 years of age [9,15,16,18,22,31,38,56]. An increased risk in mastocytosis, asthma, hypertension, coronary artery disease, obesity and morbid obesity, especially with the use of angiotensin-converting enzyme inhibitors and beta-blockers, was also reported [7,8,55]. Common chemical allergens (detergents, cosmetics, disinfectants) containing tertiary or quaternary ammonium groups induce a sensitisation state with a strong ability to cross-reations to skeletal muscle relaxants. This explains the frequent occurrence of hypersensitivity reactions to muscle relaxants during patients’ first anaesthesia experience [47,49]. Another mechanism is described in the case of pholcodine, which causes sensitisation with increased total serum IgE concentration and induces succinylcholine and morphine–specific IgE production [57–65].

## Summary

Although the first reports of perioperative hypersensitivity reaction care come from the 1990s, knowledge on this subject has grown significantly over time.

In many countries, this problem has been noticed, and teams and organisations have been established to identify and monitor the occurrence of this phenomenon. It should also be noted that there is no information about the occurrence of perioperative hypersensitivity reactions in many countries. Hence, global assessment of the problem is underestimated. The unpredictable and sudden course, the diagnostic difficulties in determining the etiologic factors, and the lack of a global assessment of the frequency of the hypersensitivity phenomenon mean that this topic is not fully understood and requires continuing research and epidemiologic follow-up to ensure the safety of the perioperative period.

In conclusion, the topic of pharmacological aspects of perioperative hypersensitivity reactions is crucial in perioperative care. The long-term epidemiologic studies have demonstrated variability in pharmacologic triggers. However, the main pharmacological substances causing perioperative (periprocedural) hypersensitivity reactions (antibiotics, muscle relaxants, disinfectants, contrast agents) are related to aspects of patient safety (anaesthesia and the prevention of post-surgical infections). Additionally, there is a great need to continue the epidemiological observations and to develop a system to monitor their occurrence in other countries.
